# Synphilin-1 Binds ATP and Regulates Intracellular Energy Status

**DOI:** 10.1371/journal.pone.0115233

**Published:** 2014-12-29

**Authors:** Tianxia Li, Jingnan Liu, Wanli W. Smith

**Affiliations:** Department of Pharmaceutical Sciences, University of Maryland School of Pharmacy, Baltimore, Maryland 21201, United States of America; Laurentian University, Canada

## Abstract

Recent studies have suggested that synphilin-1, a cytoplasmic protein, is involved in energy homeostasis. Overexpression of synphilin-1 in neurons results in hyperphagia and obesity in animal models. However, the mechanism by which synphilin-1 alters energy homeostasis is unknown. Here, we used cell models and biochemical approaches to investigate the cellular functions of synphilin-1 that may affect energy balance. Synphilin-1 was pulled down by ATP-agarose beads, and the addition of ATP and ADP reduced this binding, indicating that synphilin-1 bound ADP and ATP. Synphilin-1 also bound GMP, GDP, and GTP but with a lower affinity than it bound ATP. In contrast, synphilin-1 did not bind with CTP. Overexpression of synphilin-1 in HEK293T cells significantly increased cellular ATP levels. Genetic alteration to abolish predicted ATP binding motifs of synphilin-1 or knockdown of synphilin-1 by siRNA reduced cellular ATP levels. Together, these data demonstrate that synphilin-1 binds and regulates the cellular energy molecule, ATP. These findings provide a molecular basis for understanding the actions of synphilin-1 in energy homeostasis.

## Introduction

Synphilin-1 is a cytoplasmic protein with 919 amino acids [1]. The biological functions of synphilin-1 are not fully understood [2]. Synphilin-1 contains ankyrin-like repeats and a coiled-coil domain, and harbors predicted ATP binding motifs [1], [3]. Human synphilin-1 is expressed in various tissues and its expression is enriched in the brain [3]–[5]. Several studies suggest that synphilin-1 may be of relevance to Parkinson’s disease (PD) pathology. Synphilin-1 interacts with α-synuclein, parkin, leucine-rich repeat kinase-2 (LRRK2), other ubiquitin ligases, and proteasome subunit/regulators, and has been shown to be associated with protein aggregation [1]–[12]. *In vitro* studies have shown that the co-expression of synphilin-1 and α-synuclein promotes the formation of cytoplasmic protein aggregation [1], [6]. We and others have demonstrated that synphilin-1 has neurotrophic and neuroprotective effects [13]–[16]. Synphilin-1 protects against rotenone toxicity in a PD cell model [14] and slows down α-synuclein pathologies in a transgenic mice model [13]. Recent studies have shown that synphilin-1 transgenic mice and flies display increased triglycerides and fat deposition, as well as impaired glucose tolerance [17], [18]. Moreover, overexpression of synphilin-1 in neurons increases food intake and body weight, suggesting that synphilin-1 plays a role in energy homeostasis [17]. However, the molecular mechanisms underlying synphilin-1 neuronal protective roles and its control of energy balance are unclear.

To further investigate the cellular functions of synphilin-1, we used cellular, biological, and biochemical approaches to investigate the relationship between synphilin-1 and the cellular energy currency molecule, ATP. The results of this study not only provide a new avenue towards understanding synphilin-1-linked biology but also provide the potential molecular basis for understanding synphilin-1 roles in neuroprotection and energy homeostasis.

## Materials and Methods

### Materials

Media for cell culture and LipofectAMINE Plus reagent were from Invitrogen (Carlsbad, CA, USA). Anti-myc and anti-HA monoclonal and polyclonal antibodies were obtained from Santa Cruz Biotechnology (Santa Cruz, CA, USA). The anti-human synphilin-1 polyclonal antibody was made against the human synphilin-1 fragment (34–500 aa) and had cross-reactivity with rodent synphilin-1 as previously described [1]. Anti-actin antibody was from Sigma (St. Louis, MO, USA).

### Plasmids and site mutation

Plasmids expressing human full length myc-synphilin-1 and HA-synphilin-1 were described previously [6], [10]. The truncated HA-tagged synphilin-1 constructs were a gift from Dr. C.A. Ross and S. Engelender as described [6], [10]. The QuikChange site-directed mutagenesis kit (Stratagene, La Jolla, CA, USA) was used to change the K residue to A in the five predicted ATP binding motifs {K-x (1–5)-K} of synphilin-1 according to the manufacturer’s protocols. All of the resulting constructs were confirmed by sequencing.

### Cell culture and transfection

Human HEK293T cells and human neuroblastoma SH-SY5Y cells were purchased from ATCC. These cells were grown in Dulbecco’s modified Eagle’s medium (DMEM; high glucose; Invitrogen, Carlsbad, CA, USA) with 10% fetal bovine serum (FBS) and 1% antibiotic-antimycotic (100 units/ml penicillin, 100 µg/ml streptomycin, and 2.5 µg/ml Fungizone; Invitrogen) at 37°C under 5% CO_2_ */*95% air. Starvation was induced in the DMEM media with 2% FBS and 5 mM glucose. Transient transfections were performed with LipofectAMINE Plus (Invitrogen) according to the manufacturer’s protocol. The specific siRNA targeting synphilin-1 and its scrambled RNA control sequence were ordered from Dharmacon (Chicago, IL, USA). siRNAs were transfected into cells for 72 hours using LipofectAMINE 2000 according to the manufacturer’s protocol. Cells were harvested for Western Blot analysis or ADP/ATP measurement.

### Western blot analysis

Cells were harvested in lysis buffer as described previously [14]. The resulting lysates were subjected to Bradford protein assays to ensure equal protein loading. Cell lysates were separated with 4–12% NuPAGE Bis-Tris gels and transferred onto polyvinylidene difluoride membranes (Invitrogen). The membranes were blocked in TBST buffer (10 mM Tris·HCl, pH 7.4/150 mM NaCl/0.1% Tween 20) containing 5% non-fat milk and then probed with different antibodies. Proteins were detected by using enhanced chemiluminescence reagents (PerkinElmer, Waltham, MA, USA).

### ATP and ADP binding assays

An ATP AffiPur kit was obtained from Jena Bioscience (Jena, Germany) containing four types of ATP-agarose: Aminophenyl-ATP-Agarose, 8-[(6-Amino)hexyl]-amino-ATP-agarose, N^6^-(6-Animo)hexyl-ATP-agarose and 2′/3′-EDA-ATP-Agarose. ADP-agarose was obtained from Sigma (St. Louis, MO, USA).

ATP and ADP binding assays were performed according to the manufacturer’s protocol. Briefly, cell lysates containing 100 µg of protein for each reaction were incubated with 30 µl of ATP-agarose (or ADP-agarose) bead suspension that was pre-treated with 100 µg/ml BSA in 1×TBS buffer for 1 h at 4°C to block non-specific binding sites. After incubation for 1 h at 4°C, nucleotides (CTP, GTP, GDP, GMP, AMP. ADP, or ATP) were added to a final concentration of 2 mM, and the incubation was continued for another 2 h at 4°C. The resulting beads were washed twice with 500 µl lysis buffer, and bound protein was eluted by adding SDS–PAGE sample buffer and heating for 10 min at 72°C. Precipitates were subjected to Western blot analysis using anti-myc or anti-HA antibodies to detect synphilin-1.

### ADP and ATP Measurement

HEK293T cells were transfected with empty vector and synphilin-1 variants or siRNA targeting synphilin-1 for 72 h, and harvested in cell lysis buffer (Cell Signaling Technology). Lysates were centrifuged at 14,000 rpm for 20 min. The supernatant of each sample was subjected to Bradford protein assays. An aliquot of 10 µg of protein from each sample was subjected to ADP or ATP luminescent assays using EnzyLight ADP Assay Kit (EADP-100) (BioAssay Systems) or ATP Determination Kit (Invitrogen) according to the manufacturer’s protocol. The experiments were repeated three times in duplicate.

### Data Analysis

Quantitative data were expressed as means ± SEM based on at least three separate experiments. The differences among groups were assessed by analyses of variance (ANOVA). A *p* value of <0.05 was considered significant.

## Results

### Synphilin-1 binds with ATP

**Figure 1:**
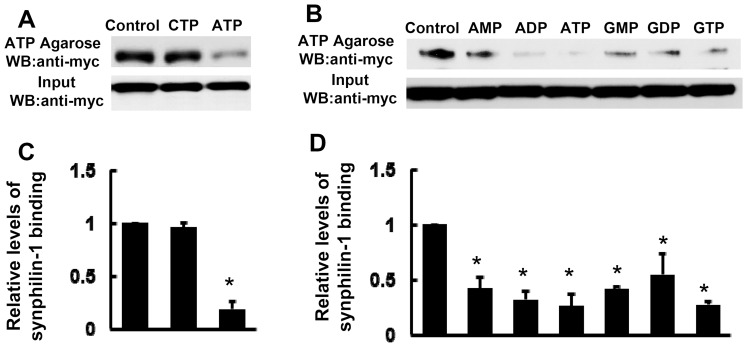
Synphilin-1 binds ATP. myc-Synphilin-1 was affinity-eluted from lysates of transfected HEK293T cells, using ATP-agarose (Jena Bioscience), in the absence or presence of CTP, AMP, ADP, ATP, GMP, GDP, or GTP at 2 mM concentration. Precipitates were resolved by SDS-PAGE and immunoblotted with anti-myc. Equal protein input was controlled by a western blot using anti-myc (bottom). A and B, Shown are representative blots from three repeated experiments. C and D, Quantification of data from A and B. *p<0.05 by ANOVA, vs control.

ATP is the major cellular molecule responsible for providing energy for various cellular processes [7]. Bioinformatic studies have shown that synphilin-1 harbors predicted ATP binding motifs, K-x (1–5)-K [1]. To test whether synphilin-1 binds with ATP, we performed ATP binding assays using ATP conjugated-agarose. ATP-agarose bound with synphilin-1 (Fig. 1A). Addition of free ATP (but not CTP) eluted the majority of synphilin-1 from ATP-agarose up to ∼90%, indicating the reversible nature of the synphilin-1/ATP binding (Fig. 1A). Furthermore, the addition of AMP, ADP, GMP, GDP, or GTP also partially eluted synphilin-1 from ATP-agarose (Fig. 1B). ADP had a similar binding efficacy to ATP, while the other tested nucleotides had relatively lower binding efficacy. GTP is also a molecule that provides energy for cellular processes. Both GTP and ATP can often transfer energy with each other by the chemical cleavage (or addition) of the phosphate bond [20]. GMP and GDP are breakdown molecules from GTP. AMP and ADP are breakdown molecules from ATP [20]. Given the similar functions between ATP and GTP, we only studied the relationship between synphilin-1 and ATP/ADP in the below experiments.

**Figure 2:**
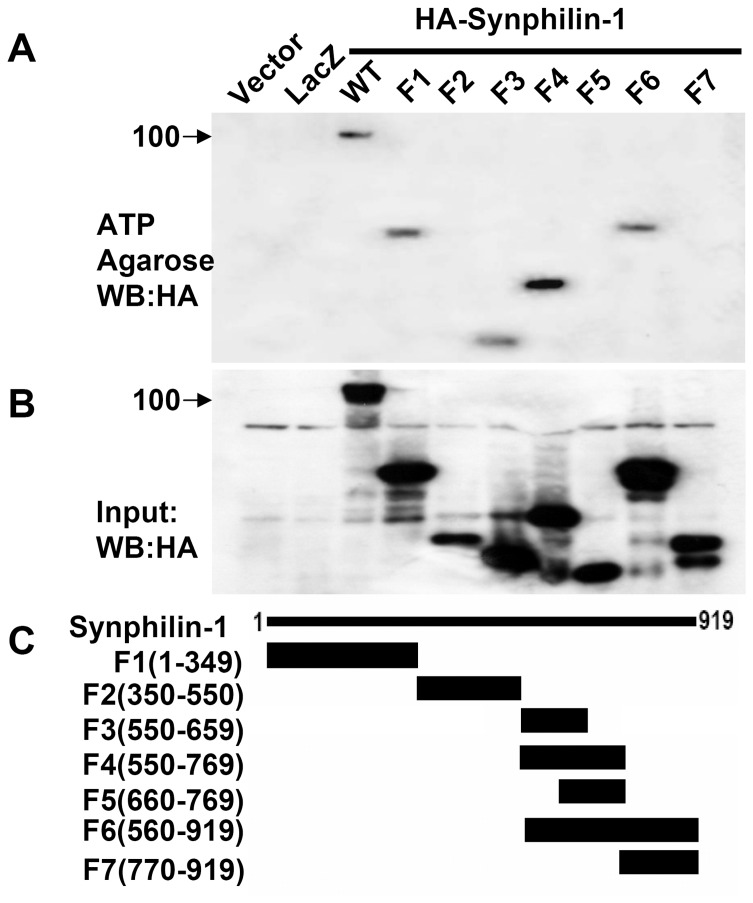
ATP binding regions in synphilin-1. HEK293T cells were transfected with various HA-tagged synphilin-1 truncated constructs for 48 h. The cells were harvested and the cell lysates were precipitated with ATP-agarose. The resulting precipitates were resolved by SDS-PAGE and immunoblotted with anti-HA antibodies. ATP bound with synphilin-1 predominantly at two regions: 109–349 aa and 550–660 aa. The blots are representative of three separate experiments. Lacz, pcDNA3.1-Lac-Z control construct. A, ATP binding synphilin-1 fragments. B, Loading control of synphilin-1 fragments. C, Diagram of synphilin-1 fragments.

To further map the ATP binding region of synphilin-1, we used various HA-tagged truncated synphilin-1 constructs to perform ATP binding assays. The F1 (1–134 aa), F3(550–659 aa), F4(550–769 aa), and F6 (560–919 aa) constructs bound with ATP as well as full length synphilin-1 protein (Fig. 2). In contrast, the F2 (350–550 aa), F5 (560–769 aa) and F7 (770–919 aa) did not bind ATP. In a separate experiment, we found that the fragment, 1–108 aa, also did not bind ATP. These results indicated ATP predominantly binds with two regions of synphilin-1 (109–349 aa and 550–660 aa).

**Figure 3:**
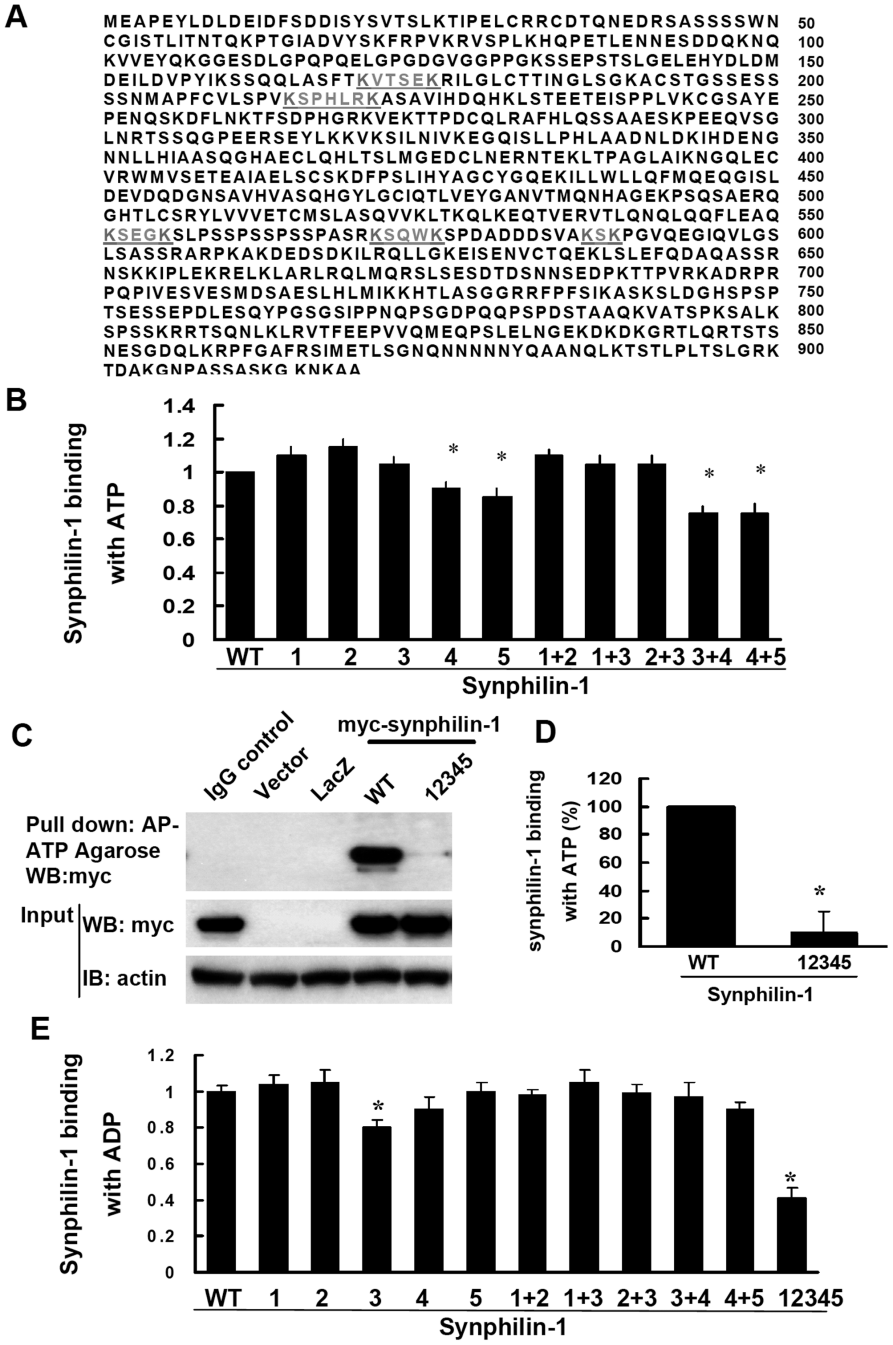
ATP binding motifs in synphilin-1. **A**. Predicted ATP binding motifs (KXXXK) of synphilin-1. Underline indicates five predicted ATP binding motifs in two regions (109–349aa and 550–660aa), from N-terminus to C-terminus, named as sites 1 to 5. The ‘Ks’ were mutated to ‘As’ by site mutagenesis. **B**. HEK293T cells were transfected with empty vector, non-related protein-Lac-Z, myc-synphilin-1, and various altered constructs of myc-synphilin-1 as indicated for 48 h. Lysates were harvested and precipitated with ATP-agarose (A–D) and ADP-agarose (E). The resulting precipitates were resolved by SDS-PAGE and immunoblotted with anti-myc antibodies. The amount of synphilin-1 binding with ATP or ADP was quantified. WT: wild type synphilin-1. 1–5 indicated synphilin-1 constructs with alterations in each of the predicted ATP binding motifs. **C** and **D**, HEK293T cells were transfected with empty vector, myc-tagged-Lac-Z, myc-synphilin-1, and myc-synphilin-1-mATP (abolishing the five predicted ATP binding sites) for 48 h. Lysates were subjected to ATP binding assays. **D**. Quantification data of **C**. **E.** Quantification data of synphilin-1 binding with ADP. The experiments were repeated three times with similar results. *p<0.05 by ANOVA, vs control.

A bioinformatic sequence match search on these two regions of synphilin-1 revealed five predicted ATP binding motifs (Fig. 3A). Using a site mutagenesis approach, we mutated the K residue to A in each of the predicted ATP binding motifs as indicated in Fig. 3A. Individual alterations of motifs 1, 2, and 3 did not alter ATP binding activity, compared to wild type synphilin-1 (Fig. 3B). Alteration of motifs 4 or 5 only slightly reduced ATP binding activity by 15–20% (Fig. 3B), while alteration of all five motifs together (synphilin-1-mATP) significantly reduced synphilin-1 binding with ATP up to ∼90% (Fig. 3C and 3D). Furthermore, we also found that synphilin-1-mATP also reduced synphilin-1 binding with ADP, but to a lesser degree, with ∼60% reduction (Fig. 3E). These results suggest that ATP and ADP share the same motifs for binding with synphilin-1 and that all five ATP binding motifs are required to form the 3D structure of ATP/ADP binding site(s).

### Overexpression of synphilin-1 increased cellular ATP levels but did not alter ADP levels

**Figure 4:**
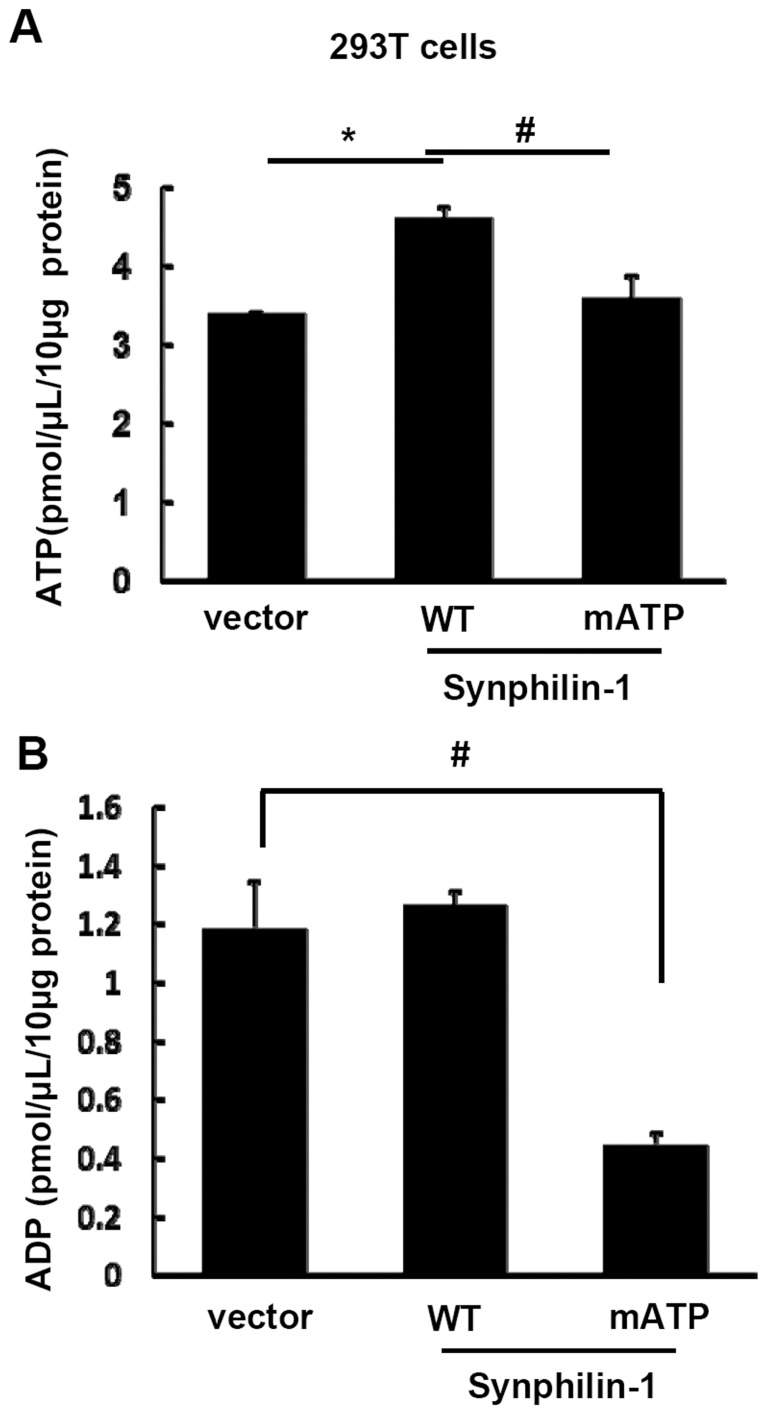
Synphilin-1 increases cellular ATP levels. HEK293T cells were transfected with empty vector, myc-human-synphilin-1, and myc-synphilin-1-mATP (abolishing the five predicted ATP binding sites) for 48 h. Lysates were harvested and subjected to ATP (**A**) and ADP (**B**) assays. The experiments were repeated three times. All data are expressed as mean ± SEM. *p<0.05 by ANOVA, significant differences between cells expressing wild type synphilin-1 and empty vector; # *p*<0.05 by ANOVA, significant differences between cells expressing wild type synphilin-1 and synphilin-1-mATP variant.

To test whether synphilin-1 alters cellular ATP content in cells, we performed an ATP luminescence assay using cell models. Transient overexpression of human synphilin-1 in HEK293T cells significantly increased cellular ATP content, while expression of synphilin-1-mATP reversed the increased ATP levels to baseline levels seen in vector control cells (Fig. 4A). In contrast, overexpression of synphilin-1 in HEK293T cells did not alter cellular ADP levels compared with vector control cells (Fig. 4B). However, expression of synphilin-1-mATP disrupted ATP binding activity of synphilin-1 and reduced the cellular ADP levels compared with vector control cells (Fig. 4B).

### Knockdown of synphilin-1 expression reduced cellular ATP levels

**Figure 5:**
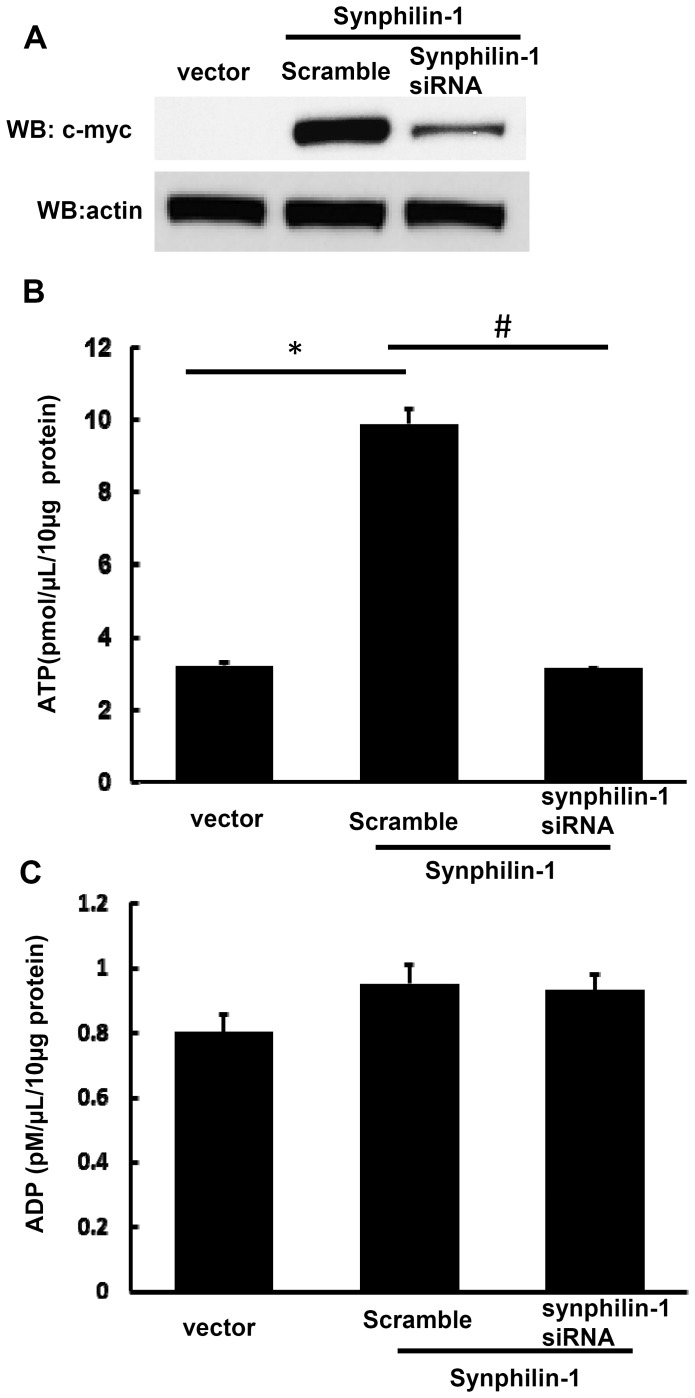
Knockdown of synphilin-1 reversed cellular ATP levels to baseline levels. HEK293T cells were co-transfected with siRNA targeting synphilin-1 and cDNA synphilin-1 constructs at 4∶1 ratio for 72 hours. Cells were harvested for Western blot analysis (A), ATP (B), and ADP assays (C). The experiments were repeated three times. All data are expressed as mean ± SEM. *p<0.05 by ANOVA, significant differences between cells expressing vector and synphilin-1; # p<0.05 by ANOVA, significant differences between scramble control siRNA and siRNA targeting synphilin-1 group cells expressing wild type synphilin-1.

To further confirm whether synphilin-1 alters cellular ATP levels, HEK293T cells were co-transfected with siRNA targeting synphilin-1 and synphilin-1 cDNA constructs at a 4∶1 ratio for 72 hours. Expression of synphilin-1 siRNA significantly reduced the synphilin-1 cDNA expression (Fig. 5A, top panel) compared with cells transfected with control scramble RNA, while knockdown of synphilin-1 overexpression by siRNA reversed cellular ATP to similar baseline levels as vector control cells (Fig. 5B). In contrast, knockdown of synphilin-1 overexpression did not alter the cellular ADP levels (Fig. 5C).

**Figure 6:**
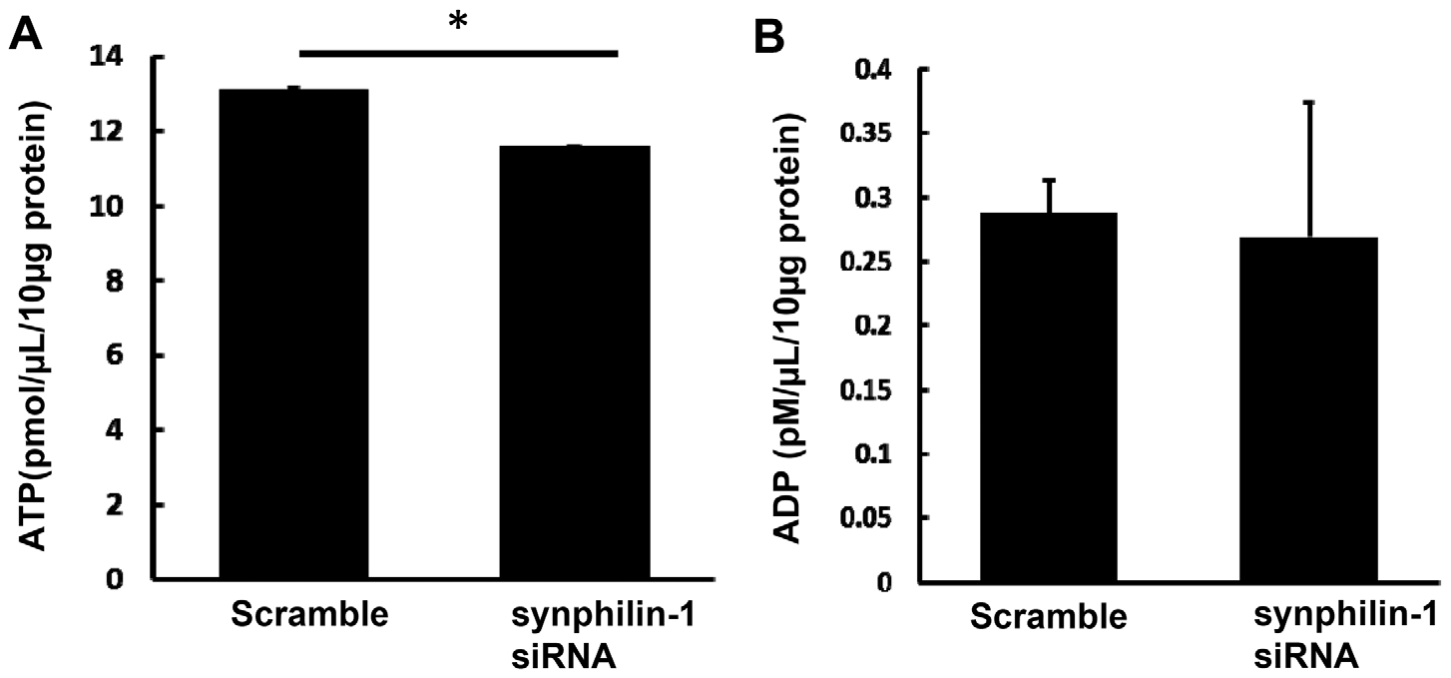
Knockdown of endogenous synphilin-1 reduced cellular ATP levels. Human neuroblastoma SH-SY5Y cells were transfected with siRNA targeting human synphilin-1 for 72 hours. Cells were harvested for ATP (A) and ADP assays (B). *p<0.05 by ANOVA, significant differences between scramble control RNA and siRNA targeting synphilin-1 groups.

To assess the roles of endogenous synphilin-1 in regulating ATP levels, human neuroblastoma SH-SY5Y cells were used, as they express endogenous synphilin-1 proteins. SH-SY5Y cells were transfected with the specific siRNA targeting synphilin-1 for 72 hours. Knockdown of endogenous synphilin-1 by siRNA reduced the cellular ATP levels compared with control (Fig. 6A). Similar to what we found in the above overexpression model, knockdown of endogenous synphilin-1 did not alter the ADP levels (Fig. 6B). Together, these data suggest that overexpression of synphilin-1 up-regulates the cellular energy currency, ATP levels.

## Discussion

The major findings of these studies are that synphilin-1 bound ATP and increased cellular ATP levels. Our results showed that synphilin-1 reversibly bound with ATP and ADP with relatively high binding efficacy, with greater ATP binding than ADP binding. Synphilin-1 also bound with other energy molecules, including AMP, GMP, GDP, and GTP with a lower binding efficacy. These findings provide the molecular basis for synphilin-1 in regulating the cellular energy. Moreover, we identified 5 motifs in two regions of synphilin-1 that were responsible for synphilin-1 binding with ATP. Interestingly, synphilin-1 increased cellular ATP levels but not ADP levels, suggesting that synphilin-1 up-regulates ATP synthesis but does not alter ATP break down, thereby elevating the cellular energy levels. Genetic disruption of ATP binding activity or knockdown of synphilin-1 expression reduced cellular ATP levels, further supporting the idea that the synphilin-1 plays a critical role in regulating the cellular energy status. To our knowledge, this is the first report that synphilin-1 bound with ATP and regulated cellular energy molecules.

ATP is the major energy currency of the cell, providing the energy needed for cellular activities within the cell, including synthesis of polysaccharides, fats, RNA, DNA, and proteins, as well as regulation of various biochemical pathways [20]. These biochemical pathways include the active transport of molecules and ions, maintaining neuronal activity, cell survival, cell growth, and adding phosphate groups to many other signaling proteins [20].

ATP is generated from catabolic pathways (such as fatty acid oxidation and glycolysis) [19] through the addition of a phosphate group to ADP. This process stores energy in ATP. When ATP breaks down to ADP or AMP and an inorganic phosphate, this energy is released via cleavage of the chemical bond between the second and third phosphate in ATP [20]. Thus, the ATP/ADP cycle in cells acts to store and provide energy necessary to maintain proper cellular functions and energy homeostasis. Our results showed that synphilin-1 bound ATP and increased cellular ATP levels. Although synphilin-1 also bound ADP to a lesser extent than ATP, it did not alter ADP levels. Knockdown of endogenous synphilin-1 in SH-SY5Y cells reduced cellular ATP levels but did not alter ADP levels. These findings suggest that synphilin-1 fosters ATP synthesis without altering the ATP metabolic pathway, thereby positively regulating the cellular energy balance. Further investigation remains on whether synphilin-1 fosters ATP synthesis via regulating cellular signaling pathways resulting in upregulation of energy producing processes given that synphilin-1 has been shown previously to activate extracellular signal-regulated kinase (ERK1/2) signaling pathways [14].

The increase in ATP levels inside the cells could result in two types of cellular changes. One is activation of the energy storage pathways to promote glycogen and lipid synthesis that may ultimately induce fat and glycogen accumulation and deposition [20]. In line with this type of change, our previous studies have shown that overexpression of synphilin-1 increases triglyceride levels and fat storage both in mice and in flies, as well as induces insulin resistance [17], [18]. The obese-like phenotypes in synphilin-1 mice and flies more likely represent the consequence of synphilin-1 up-regulating cellular energy currency, ATP levels. The other type of change involves in providing ample energy for various cellular functions including cell growth and protection against toxic stimuli [20]. Previous studies have shown that synphilin-1 has a neurotrophic effect and protects against rotenone and α-synuclein toxicity in PD models [1], [3], [5]–[7]. Increases in cellular ATP levels may be one of the potential molecular mechanisms for neuronal protection roles of synphilin-1 by providing more energy to synthesize protective proteins and clear toxic stimuli. In addition, increases in cellular ATP levels may also facilitate the interactions between synphilin-1 and other proteins (eg. α-synuclein and parkin) in forming protein aggregates [1], [3], [5]–[7] to help the sequestration or clearance of toxic proteins from the cytosol.

In summary, our findings reveal novel cellular functions of synphilin-1 in regulating ATP levels, that provides a new molecular basis for understanding synphilin-1 biology and the molecular mechanisms for synphilin-1 roles in neuronal protection and energy homeostasis.
